# Facts Concerning Physical Condition of Women during College Life1Read before Women’s Medical Society of New York State at its 6th Annual Meeting in Buffalo, May 10, 1912.

**Published:** 1912-07

**Authors:** Esther E. Parker

**Affiliations:** Physical Examiner and Medical Adviser of Women of Cornell University; 326 East State Street, Ithaca, N. Y.


### Facts Concerning Physical Condition of Women During College Life[1]

By ESTHER E. PARKER, M.D.,
Physical Examiner and Medical Adviser of Women of Cornell University
326 East State Street, Ithaca, N. Y.

Unfortunately, the statistics I have to present to you today are fragmentary. Although I wrote for data to thirty universities and colleges, I received replies from about ten. In many cases, statistics were not compiled, but valuable observations, which are often more reliable than statistics, were sent.

THE office of Medical Adviser, as distinct from that of Physical Examiner, for women at Cornell University has been inaugurated this year. For two years such provision has been made for the men.

In my capacity as Medical Adviser, I have come in close contact with the women, holding, as I do, office hours at the University each day and issuing all their excuses for absences.

I was first surprised at the large number who reported dys-

1 Read before Women's Medical Society of New York State at its 6th Annual Meeting in Buffalo, May 10, 1912.

This is not statistical but an observation by the gymnasium instructor.

*Northwestern*:

Irregularities few.
Dysmenorrhoea, not serious.
Amenorrhoea, 2 or 3 out of 180-200.

*Wells College*:

Majority regular.
Dysmenorrhoea few.
Amenorrhoea few.

*Syracuse University*:

"Medical examinations of all entering girls show general dysmenorrhoea, which gradually disappears during the first year of college. I find then a tendency toward monorrhagia and the menses occurring every three weeks. There have been but few cases of amenorrhoea. There are, however, two cases in college at present, both attending special classes in the gymnasium. The menses in one case occurred before the end of the freshman year and the other in the senior year. Both girls are in good health. There are a few cases of irregularity the periods occurring every five or six weeks. These girls are in a special outdoor walking class."

The replies to the question regarding "nervous disturbances," were very satisfactory in that so few are found. At Tulane University, an exception is found and Miss Baer writes:

Anorexia, small percentage.

Insomnia, small percentage.

General nervous temperament, large percentage.

Examinations are the dragons causing insomnia, and in the spring anorexia is most prevalent.

When I spoke to Dr. Tinker, who has done such splendid work on goitres, about the astonishingly large number found among the entering women this year, he said he felt sure that it was not that goitre is more common but that it is being noticed more and more. Out of 184 cases examined, I found 27 that I feel sure of and 7 doubtful ones.

From Western Reserve, Miss May writes the "number of cases is appalling."

At Smith there are 53 out of 892.

At Mt. Holyoke, 3 per cent.

At Brown 1 case (out of 50?)

At Radcliffe, 9 out of 401.

At Northwestern, about 20.

At the University of Indiana, 10-15 out of 250.

At Wells, 3 cases.

At Syracuse University, 1909, 11 out of 301; 1910, 1 out of 313; 1911, 4 out of 307.

At Tulane University, very rare.

At University of Illinois, no statistics, occasional cases.

Have you heard the small boy's excuse for not washing his back sufficiently often? When his mother questioned him closely about it he admitted a lack of attention to this part of his anatomy with the convincing remark "you know, mother, I don't use it much." The backs I have seen this year prove beyond a doubt how very much and how poorly they are used. If we could induce mothers not to corset their daughters so early, and to be more careful about the size of the heels on their shoes; if we could influence school superinendents to have school desks adjustable to the size of the student and properly lighted; and if growing muscles were given freedom of movement instead of being trained to keep still, we could then hope to reduce the number of spinal curvatures, which is far too large.

More than one-third of the women I examined last fall showed curvatures varying from slight to marked cases. The fact that they are, for the most part, postural, does not make them any the less a deformity.

From Western Reserve, Miss May writes optimistically: "The spinal curvatures are noticeably decreasing in numbers, owing to the fact, I feel sure, that physical training is better taught and taught in more preparatory schools that ten years ago. The serious cases are few."

M. Holyoke reports 20-25 per cent.

Brown reports 14 out of 15 freshmen.

Radcliffe reports 20 per cent.

Northwestern reports not uncommon.

University of Indiana reports 70 per cent.

Wells reports about 33 1-3 per cent.

Syracuse reports 37 out of 301 in 1909.

Oberlin, Dr. Hanna reports: Scoliosis, 58 3-7 per cent; Kyphosis, 16 2-3 per cent; Lordosis, 4 2-27 per cent; faulty posture, 5 25-27 per cent.

Tulane University reports 85-90 per cent.

University of Illinois reports 51 per cent.

The statistics for weak arches and flat foot are:

Cornell, a little more than 10 per cent have weak ankles or flat foot.

menorrhoea; many were obliged to remain in bed an entire day, more but half a day, and a few for two days. Realizing that there was a large number of women who were not asking for excuses, and desiring to find out more about the effect college was having upon menstruation, I called a mass meeting and asked the girls to fill out cards for me. In a busy university community, where the demands upon a student's time are so many and so varied, it is almost impossible to arrange a meeting when a large majority of the students can attend. I was able, however, to get these cards filled out by 243 women. The usual questions concerning menstruation were asked, i. e., age at which established, regularity, details if irregular, amount, duration, pain; detail as to location, severity, character; other symptoms, headache, nervousness, nausea, etc. In addition, I asked that they make special note of any difference observed during college term and summer vacation; whether they gained or lost weight in college and whether or not they wore glasses.

In this way I secured statistics from upper class women of whom we have no records in the gymnasium since their Sophomore year. Gymnasium work is required of all Freshmen and Sophomores, unless they are found unable to take it; histories are taken of entering students and physical examinations made in the fall term of the Freshmen and Sophomore years.

The large majority of our students enter in good condition. Out of 184 examined this fall, I classified 24 in excellent health, 97 good, 48 fair, and 15 poor. Under the heading "poor" I included those showing nervous conditions, markedly defective metabolism, and in one case a serious cardiac lesion and large goitre. It is worthy of note that many of these classified as poor have finished the year in much better condition than some who have been in apparently better health and less nervous.

From the cards which 243 women filled out, I gathered the following data on general weight during the college course: 36 lost; in some cases this loss was the direct result of dieting undertaken with the purpose of losing excess fat, in others the amount lost is reported slight and in several the remark is made "lost weight during college, but general health is better than before, appetite keener and less nervous." A large number of our women live at home or board down town and walk to and from the campus. This necessitates a steady climb no matter from what direction the campus is approached, for, as we sing in our Alma Mater, Cornell is "Far above Cayuga's waters and its waves of blue." Fifty-eight students reported the same weight and 149 gained. The gain was in most cases quite marked, from four to ten pounds, and, in one case, thirty-five.

This is not at all unusual, judging from reports from other universities and colleges.

At Smith, statistics for the end of the year were not yet completed, but out of 892 students examined in the fall, 743 were classified as good or excellent. Dr. Underhill wrote from Mt. Holyoke, that out of 1,141, 93.6 per cent maintained their weight or improved. From Brown University, Dr. Arnold says, "There is a notable loss in the first year." Statistics from Radcliffe show a tendency to increase. At Wells College, where the great majority of the students live in dormitories, Miss Douglass writes "a good majority gain weight and maintain it throughout their four years." A the University of Syracuse, there is a general improvement noticed. Miss Clara Baer, who has charge of the gymnastic work at Tulane University, feels that "so far the general nutrition of the college girl is not so good at the end as at the beginning of the college course. There are a few exceptions to this rule; not enough, however, to feel that the college is as yet fulfilling its mission in securing to the student a more efficient organism as a whole." At the University of Indiana, where the end results are not so good, Dr. Bowers writes "the boarding clubs are abominable and many girls grow disgusted with that fare and resort to trying to exist from a paper bag. They do not get the good and nourishing food they need when doing hard mental work and many are dragged out, bilious, nervous and hysterical at the end of the senior year." Dr. Bowers goes on to say that perhaps much of this is due to injudicious use of sweets, peanuts, etc., and the many indigestible spreads the girls have. Another factor is the whirl of society.

From Western Reserve Miss May writes: I find the demands on a college student living in a city and attending college in that city are much greater than on the student who attends college in a more secluded, quiet place and who has practically only the campus life. Having come here after several years of teaching at Vassar, I was impressed with the fact that I could not expect as much from the students of the college for women, physically, as I had been getting from the students at Vassar. This, I feel, is greatly owing to the strain of long car rides to and from college, home duties and the many social duties besides those necessary in the college life."

To return to Ithaca—I felt very sure before looking over my cards that the percentage of menstrual irregularities was very large, and the figures proved it; 79 out of 243 were irregular. The variations are in one case every 10 days to 6 weeks, in one 6-12 weeks, one 24 to 31 days, several 4-6 months. Frequent menstruation is almost always traceable to overwork, either physical or mental. Some few students menstruate irregularly during the college year and become regular in the summer, while with others the opposite is true. A few who were irregular before entering college are regular now."*

Of those who menstruate regularly, there seems to be no limit to the length between periods; every 3 weeks, 25 days, 5 weeks, 32 days, 3 months, 50 days, etc. One hundred and thirty have pain which amounts to more than an uncomfortable feeling or general weakness. Of these, 62 have moderate pain, 29 slight and 39 severe; 54 report severe headache, general depression, nausea or backache.

There are two cases in which the menstruation has not yet been established and four cases of temporary amenorrhoea. One student has not menstruated in four and another not in nine months.

Statistics on menstruation, from other colleges are:

*Western Reserve*:

Menorrhagia, prevalent.
Dysmenorrhoea, noticeable few.
Amenorrhoea, none reported.

*Smith College*:

130 out of 892, irregular.
41 out of 892, dysmenorrhoea.
68 out of 892, amenorrhoea.

*Mt. Holyoke*:

Out of 1,141 there are disturbances in 280. Of these
56.4 per cent improved.
41.4 per cent about same.
2.2 per cent grew worse.
Amenorrhoea, about ⅝ per cent freshmen.

*Brown University*:

Almost no cases of Dysmenorrhoea.
Amenorrhoea, few temporary cases.

*Radcliffe*:

Out of 401:
6, too profuse.
6, too frequent.
3, Dysmenorrhoea severe.

---

*Not enough figures for statistics.

Western Reserve, "many weak ankles, few flat feet."
Smith, no statistics.
Mt. Holyoke, 20 per cent.
Brown, rather common.
Radcliffe, comparatively few flat feet, many weak feet.
University of Indiana, 75-77 per cent.
Wells College, 6 not bad.
Oberlin, 64 4-9 per cent.
Tulane, 20 per cent.
University of Illinois, about 75 per cent.

Defective vision is so universal that statistics only repeat the well known fact. Out of 89 cases examined, I found 71 with defects. Statistics from other universities are:

Mt. Holyoke, 50 per cent.
Brown, common.
Radcliffe, many cases.
Syracuse, in 1910, 80 out of 305; in 1911, 99 out of 268.
Tulane, 20 per cent.

As to results of gymnasium work, there is not a question of doubt; the universal answer is "improvement." Much attention is being given to corrective work and enthusiasm stimulated by its great success.

Our hope for the future is that thorough courses in physiology and hygiene may be given in the grade, grammar and high schools by physicians who are especially adapted to this work. When we teach our boys and girls a sensible knowledge of their bodies and how to take care of them, then we may hope to find the majority of men and women in our universities and colleges more nearly approaching a perfect type of beauty both physical and mental. The public health movement will surely solve the difficulties of the future by educating the general public to the needs of the coming generation.

We may feel justly proud of our honorary president, whose work along these lines has been so noteworthy.

